# High-Efficiency Enrichment of Megakaryocytes and Identification of Micromegakaryocytes from Human Bone Marrow by Imaging Flow Cytometry

**DOI:** 10.3390/cells14080588

**Published:** 2025-04-12

**Authors:** Maya Nautrup Pedersen, Trine Engelbrecht Hybel, Jens Haugbølle Bjerre, Anne Sofie Borg Hammer, Anja Bille Bohn, Marie Bill, Carina Agerbo Rosenberg, Maja Ludvigsen

**Affiliations:** 1 Department of Hematology, Aarhus University Hospital, 8200 Aarhus, Denmark; mayapd@rm.dk (M.N.P.); trihyb@rm.dk (T.E.H.); marie.bill@rm.dk (M.B.); majlud@rm.dk (M.L.); 2 Department of Clinical Medicine, Aarhus University, 8200 Aarhus, Denmark; 3 Department of Pathology, Aarhus University Hospital, 8200 Aarhus, Denmark; jensthom@rm.dk; 4 Department of Pediatric and Adolescent Medicine, Aarhus University Hospital, 8200 Aarhus, Denmark; annham@rm.dk; 5 Department of Biomedicine, FACS Core Facility, Aarhus University, 8000 Aarhus, Denmark; anja@biomed.au.dk

**Keywords:** imaging flow cytometry, megakaryocytes, micromegakaryocytes, megakaryopoiesis, human bone marrow, myelodysplastic syndromes, myelodysplastic neoplasms, artificial intelligence, convolutional neural network, deep learning

## Abstract

Megakaryocytes (MKs) are rare, large, polyploid bone marrow (BM) cells responsible for the production of platelets. The identification and characterization of MKs is widely recognized as challenging. Manual microscopy is especially difficult due to the rarity and complex morphology of MKs, while flow cytometry faces additional challenges from MKs’ large size, fragility, and platelet adhesion, causing false positives. We present a novel approach to accurately enrich MKs from human BM aspirates with a specific focus on the detection and quantification of microMKs. By integrating CD41^+^ cell enrichment, immunophenotyping, and morphometric analysis, we identified cells of the megakaryocytic lineage. To increase accuracy, a convolutional neural network was trained to identify CD41^−^ cells falsely displaying an MK-like immunophenotype due to adhesive CD41^+^ platelets. This allowed for exclusion of 94.9% of false positive events, considerably enhancing specificity. CD41 positive enrichment prior to imaging flow cytometry acquisition increased the MK frequency nearly 200-fold, yielding a population of both mature and immature MKs, thus supporting analysis of MK progenitors. Overall, this advanced approach enables enrichment of MKs from human BM, considerably increasing the accuracy and statistical power of the MK analysis. This may provide an important addition in the context of MK-related diagnostics and research.

## 1. Introduction

Megakaryocytes (MKs) are specialized bone marrow (BM) cells responsible for the production of platelets and for regulating hemostasis and thrombosis [1]. They develop from pluripotent hematopoietic stem cells (HSCs) [2] and are the largest (50–100 μm) as well as one of the rarest cell types in the BM, accounting for only 0.01–0.05% of the nucleated cells [2,3]. As MKs mature, they undergo a distinct process known as endomitosis, in which they replicate their DNA without dividing, leading to large cells with ploidy levels reaching up to 128N [2,4,5]. In humans, MK identification may be aided by antibodies targeting essential platelet membrane glycoproteins, such as CD41a, CD61, CD42b, CD36, and von Willebrand factor [3]. The most commonly targeted antigens are CD41 and CD61 [3,4,5,6], forming a heterodimeric receptor complex in the cell membrane of MKs [7]. These antigens are expressed throughout megakaryopoiesis, from progenitor cells to mature platelets, with the expression levels increasing as MK differentiation progresses [6,8].

Several conditions can lead to disturbances, dysfunction, or disrupted development of MKs. One such condition is myelodysplastic neoplasms (MDS), a heterogeneous group of clonal BM disorders defined by cytopenia and morphologic dysplasia [9], often linked to genetic abnormalities and an increased risk of progression to acute myeloid leukemia [9,10,11,12,13]. In addition to megakaryopoiesis, other BM cell lineage maturation processes may be affected, i.e., granulopoiesis and erythropoiesis [9]. In the absence of MDS-defining genetic abnormalities, the diagnostic hallmark of MDS is recognition of dysplastic features in >10% of the cells in one or more of the myeloid cell lineages [9]. Although diagnostic criteria for MDS are well-established, recognizing morphological changes can be challenging, particularly in cases with marginal dysplasia. Ambiguous findings in blood and BM, along with hypocellularity or fibrosis, further complicate the diagnosis and classification of MDS. These challenges frequently lead to results marked by inter-observer variability, even among experienced hematopathologists [14,15,16,17]. Evaluating MK morphology is often especially difficult. Although European LeukemiaNet and WHO guidelines recommend assessing at least 30 MKs in BM smears [18,19,20,21], this can be difficult to achieve due to the scarcity, fragility, and tendency of MKs to aggregate [4].

Flow cytometric immunophenotyping is a rapid, sensitive, and high-throughput technique well-suited for single-cell analysis of heterogeneous populations, including rare cell types. Abnormal immunophenotypes and atypical differentiation patterns have been shown to indicate dysplasia in granulocytic, monocytic, and erythrocytic lineages [22,23,24,25,26,27,28,29,30,31]. While flow cytometry has also been investigated for MK analysis [3,5,32], it is widely recognized that, in the context of MDS, MKs present technical challenges for flow cytometric assessment. These challenges stem from their low frequency, large size, fragility, and the tendency of platelets to adhere to non-MK cells, causing those cells to display an MK immunophenotype [24,33]. Additionally, the few MK surface markers commonly accepted are not exclusive to MKs [34].

Among the various types of morphological dysplasia in the BM, dysplastic MK features, such as micromegakaryocytes (microMKs), are strongly associated with MDS [20,35,36]. Nevertheless, dysplasia is not limited to MDS patients; it can also be observed in individuals with, e.g., nutritional deficiencies and infections, and is even present in BM from healthy individuals [20,36,37]. This highlights the importance of reliable identification and characterization of this cell lineage. Interestingly, previous studies have demonstrated the effectiveness of imaging flow cytometry (IFC) in identifying and quantifying MKs and their progenitors from heterogeneous murine BM [38,39,40]. In this study, we present a novel approach that integrates IFC with deep learning for the precise identification of microMKs aimed at overcoming the limitations of traditional morphological assessments. Specifically, IFC combines the high-throughput and statistical rigor of conventional flow cytometry with the spatial resolution of microscopy, capturing detailed images of each cell as it flows through the cytometer [41]. These images provide extensive morphometric data, including measurements of cell size, shape, texture, and the expression of fluorescently labeled markers. By incorporating both immunophenotyping and morphometrics, we developed a standardized and automated analysis protocol that accurately identifies and quantifies microMKs. Additionally, the use of a convolutional neural network (CNN) for image classification provided a critical quality control step in the gating process, effectively minimizing false positive (FP) MK identifications caused by platelet adhesion to non-MK cells. This advanced flow cytometry-based method has the potential to enhance the statistical power of microMK analysis by enabling the evaluation of a significantly larger population of MKs from BM aspirates, thereby providing a more reliable and thorough diagnostic tool.

## 2. Materials and Methods

### 2.1. Bone Marrow Samples

In total, BM samples from 11 patients undergoing diagnostic evaluation of cytopenia were included in this study. This included two MDS patients, two clonal cytopenia of undetermined significance (CCUS) patients, one chronic myelomonocytic leukemia (CMML) patient, one aplastic anemia patient, three pathological controls, and two patients for whom a definitive diagnosis had not yet been established. Nine of these patients (ID2–10) were enrolled after providing written informed consent, while the final two patients (ID1 and ID11) were included as part of quality control and method optimization (Table 1). All samples consisted of fresh excess material collected from patients as part of the clinical diagnostic process at the Department of Hematology, Aarhus University Hospital (AUH), Denmark (DK). The samples were used for one or more of the following purposes: (I) testing the effects of CD41 positive selection, (II) morphological examination of the enrichment procedure, and (III) development of an IFC panel and gating strategy for MK identification. This study was approved by the Danish Data Protection Agency (record no.: 1-16-02-849-17) and the Central Denmark Region Committee on Health Research Ethics (record no.: 1-10-72-125-17) and conducted in accordance with the Declaration of Helsinki.

### 2.2. Selection of CD41^+^ Cells

**Figure 1:**
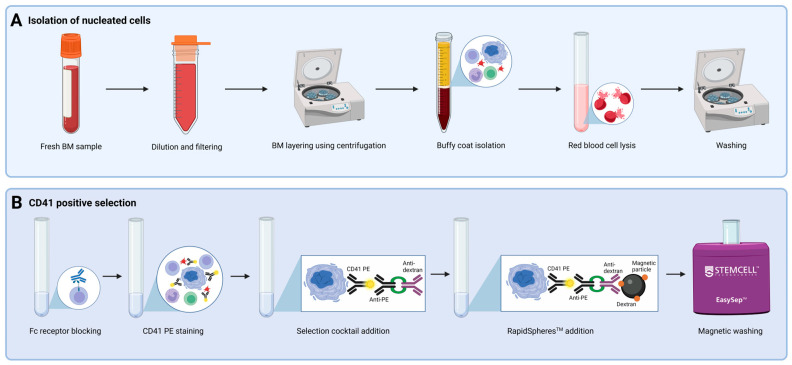
Overview of CD41^+^ cell selection. Steps incorporated in CD41 positive selection, including (**A**) isolation of nucleated cells and (**B**) selection of CD41^+^ cells using the EasySep^TM^ Human PE Positive Selection Kit II. Abbreviations: BM, bone marrow;; PE, phycoerythrin. Created using Biorender.com. (1 November 2024).

Fresh BM samples were diluted five times with sample buffer (SB) containing Dulbecco’s phosphate buffered saline (ThermoFisher, Waltham, MA, USA) with 2% fetal calf serum (Biowest, Nuaillé, France). Large blood aggregates and clumps were removed either manually or by filtering through a 300 μm filter (PluriSelect, Leipzig, Germany) to accommodate the large size of MKs. Separation of the buffy coat from plasma and red blood cells was conducted by centrifugation for 10 min at 800× *g* with no brake. Next, the buffy coat was isolated by pipetting. Residual erythrocytes were removed by addition of lysis buffer (Amplicon, Odense, Denmark), followed by incubation for 15 min at room temperature (RT) and washing with CellWash (BD Biosciences, Franklin Lakes, NJ, USA). Cellular material was reserved for an unstained control and relevant compensation samples. Positive selection using the EasySep^TM^ Human PE Positive selection Kit II (STEMCELL Technologies, Vancouver, BC, Canada), EasySep^TM^ magnets (STEMCELL Technologies), and CD41-phycoerythrin (PE) antibody (BioLegend, San Diego, CA, USA) was performed according to the manufacturer’s protocol. In short, the cell concentration was adjusted to 100 × 10^6^ cells/mL, Fc receptors (FcRII/CD32) were blocked with Anti-Human CD32 FcR Blocker (STEMCELL Technologies), and the cells were stained with the CD41 PE antibody for 15 min at RT. Next, EasySep^TM^ PE Selection Cocktail (STEMCELL Technologies) containing anti-PE and anti-dextran antibodies was added, and the sample was incubated for 10 min at RT. RapidSpheres^TM^ (dextran-coated magnetic particles, STEMCELL Technologies) were vortexed and added to the sample, which was then incubated for 10 min at RT. Three rounds of magnetic washing using RoboSep^TM^ buffer (STEMCELL Technologies) were performed to remove CD41^−^ cells. Isolated CD41^+^ cells were resuspended in RoboSep^TM^ buffer. A schematic overview of the CD41^+^ cell selection procedure is shown in Figure 1.

### 2.3. Morphological Examination of CD41 Positive Enrichment Efficiency

A BM aspirate smear from patient ID10 was created as part of the diagnostic process at the Department of Hematology and Department of Pathology, AUH, Denmark. Additionally, after CD41^+^ cell selection, a small aliquot of the resulting cell solution was centrifuged onto a slide (Hounisen Lab Equipment A/S, Skanderborg, Denmark). Both slides were Giemsa stained using a Hematek^®^ 3000 System (SIEMENS Healthineers, Erlangen, Germany) and morphologically examined by an expert pathologist to evaluate the quality of CD41^+^ cell selection. The fraction of MKs in the diagnostic sample and the enriched CD41^+^ cell sample were estimated and compared.

### 2.4. Evaluation of CD41 Positive Enrichment Efficiency Using IFC

Bone marrow from patient ID1 was divided into three fractions that were processed as follows: (I) unfractionated, (II) buffy coat isolation, and (III) buffy coat isolation plus CD41 positive selection. All samples were stained with the viability dye Zombie Green (ZG), incubated for 15 min at RT, and washed. Next, all samples were stained with pre-titrated monoclonal anti-CD45 Krome Orange (KrO), while the unfractionated (I) and buffy coat isolated (II) samples were also stained with pre-titrated monoclonal anti-CD41 PE (Table 2). The samples were incubated for 15 min at RT, washed, and fixed using Fixation and Permeabilization Solution (BD Biosciences) to allow overnight storage. This was followed by incubation for 20 min at 4 °C, two washes with SB, and overnight storage at 4 °C. Prior to acquisition, all samples were stained with DRAQ5, incubated for 20 min at 4 °C, and washed once. The percentage of MKs was estimated in each fraction based on the acquired IFC data.

As a supplemental experiment, using BM from patient ID11, we evaluated each step of the enrichment procedure to assess the efficiency of CD41 enrichment and to confirm the isolation of MKs. As previously described, MK antigens are expressed at increasing levels during MK differentiation [6,8]. To prevent pixel saturation of the largest and most mature MKs, the sample was stained and acquired using a reduced CD41 PE antibody concentration (1.5 μg/mL) and a reduced 488 nm laser power (5 mW). Specifically, the BM was filtered using a 300 μm filter (PluriSelect) to remove large aggregates, and erythrocytes were removed via lysis buffer treatment (Amplicon). Positive selection was performed as previously described in Section 2.2 using the EasySep^TM^ Human PE Positive selection Kit II (STEMCELL Technologies). After each round of magnetic washing, the supernatant was retained for subsequent analysis to determine the MK enrichment efficiency. Isolated CD41^+^ cells, which were resuspended in RoboSep^TM^ buffer, were stained with antibodies and dyes included in the IFC-MK panel (Table 3). The CD41 positive enriched sample and supernatants were then acquired as outlined in Section 2.6. Evaluation of MK frequency was performed through visual identification and tagging of MKs.

### 2.5. Staining Protocol for the MK-Specific IFC Panel

A five-color IFC-MK panel was designed to enable identification of MKs among the CD41^+^ cells selected from BM samples. The panel was applied to patients ID2–11 and included a total of eight fluorescent markers: CD41 PE used for CD41^+^ cell selection, the pan-leukocyte marker CD45 StarBright Violet (SBV) 515, and CD3, CD19, CD15, and CD64, all conjugated to Brilliant Violet (BV) 605 and combined in a dump channel to exclude T and B cells, granulocytes, and monocytes from the analysis. Moreover, DRAQ5 and Zombie NIR^TM^ (ZN) Fixable Viability dye were included for DNA staining and the exclusion of dead cells, respectively. Isolated CD41^+^ BM cells were stained with the pre-titrated monoclonal antibodies and dyes (Table 3), incubated for 30 min at 4 °C in the dark, and washed twice using SB. To complement the use of BV-conjugated antibodies, 10 μL of BD Horizon^TM^ Brilliant Stain Buffer Plus (BD Biosciences) was added to the samples.

### 2.6. IFC Configuration and Acquisition

All samples were acquired on the ImageStream^®X^ Mk II (ISX) (Cytek Biosciences, Fremont, CA, USA) at the FACS Core Facility, Aarhus University (AU), Denmark. A data acquisition template was created in the INSPIRE^®^ software (V200.1.620.0, Cytek Biosciences). To ensure a focus on microMKs, the following laser power settings were selected: 405 nm, 120 mW; 488 nm, 200 mW; 561 nm, off; 642 nm, 150 mW; 785 nm, 1 mW. This approach does not enable the inclusion of large MKs, as their images exhibit pixels saturation at varying degrees in Ch03 at the applied 488 nm laser power, necessitating their exclusion from data analysis. Brightfield (BF) images were captured in Ch01 and Ch09, and darkfield/side scatter (SSC) signal was detected in Ch06 (IFC-MK panel) or Ch12 (evaluation of CD41^+^ cell enrichment). Each antibody/stain was detected as follows: CD41 PE in Ch03, CD45 SBV515 or CD45 KrO in Ch08, dump channel antibodies CD3 BV605, CD19 BV605, CD15 BV605, and CD64 BV605 in Ch10, DRAQ5 in Ch11, and viability dyes ZN or ZG in Ch12 or Ch02, respectively. Raw image files (.rif) were obtained at low speed and at either 40x magnification (field of view: 60 μm, pixel size: 0.5 μm; ID2–11), or 60x magnification (field of view: 40 μm, pixel size: 0.33 μm; ID1). The number of replicas acquired per BM sample varied based on the amount of available sample material. A maximum of 50,000 singlets were collected and saved for each .rif.

As samples were acquired on different days, the ISX instrument performance was validated prior to acquisition of experimental samples utilizing Rainbow Calibration Particles (Spherotech, Lake Forest, IL, USA). The median fluorescence intensity of the seventh peak was measured and compared to an established baseline interval. Fluorochrome stability was assessed by comparing fluorochrome emission spectra obtained when the antibodies were put into operation and after three months of use. The test was performed using the ID7000^TM^ Spectral Cell Analyser (SONY Biotechnologies, San Jose, CA, USA) and the associated ID7000 Acquisition and Analysis Software (V2.0.2, SONY Biotechnologies) at the FACS Core Facility, AU, Denmark.

Single-stained compensation controls were created using Quantum^TM^ Simply Cellular^®^ anti-Mouse IgG beads (Bangs Laboratories Inc., Fishers, IN, USA) for all antibodies. For the DNA dye and the viability dyes, compensation samples were created using cells. All compensation samples were incubated for 30 min at 4 °C and washed. The INSPIRE^®^ compensation wizard was utilized to collect 2000 non-saturated, positive singlets from each compensation sample. Subsequently, a compensation matrix was created in the IDEAS^®^ software using the best fit linear regression method and applied to each acquired .rif file prior to analysis.

### 2.7. Gating Strategy

**Figure 2:**
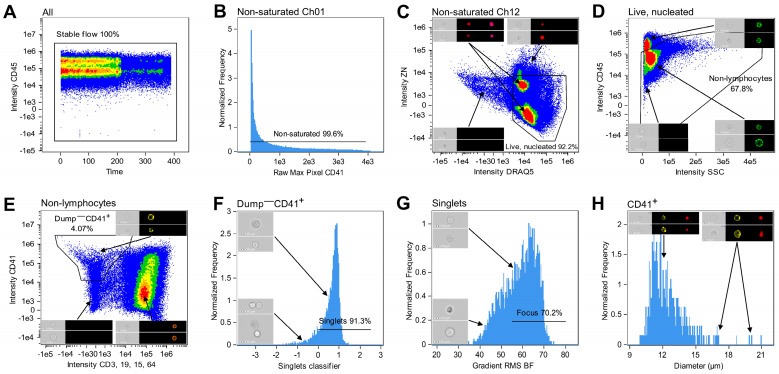
Gating strategy for identifying cells of the megakaryocytic lineage. (**A**) Events in stable flow were gated based on the distribution of cells over time. (**B**) Saturated events in all channels were excluded by only gating non-saturated events with a Raw Max Pixel value below 4030 (illustrated for the CD41 detection channel). (**C**) Using a bivariate plot of ZN and DRAQ5 intensities, live and nucleated cells were identified as being negative or low for ZN as well as positive for DRAQ5. (**D**) Lymphocytes were excluded in a bivariate intensity plot with CD45 and SSC. (**E**) Cells positive for the MK lineage marker CD41 and negative for all markers in the dump channel (CD3, CD19, CD15, and CD64) were identified and selected in a bivariate plot of the CD41 and CD3, CD19, CD15, CD64 intensities. (**F**) An ML classifier was trained to distinguish between single cells and doublets. Subsequently, using a histogram of classifier-specific values assigned to the images, singlets were differentiated from doublets and selected as having a value ≥0. (**G**) The Gradient RMS BF feature and visual investigation of histogram bins were used to gate images in focus. (**H**) Histogram displaying the diameters of the CD41^+^ population following the CNN classification. When relevant, representative images are included in the plots, showing Ch01 BF images along with fluorescence images in the channels of interest. Percentages indicate the fraction gated from the input population. Abbreviations: BF, brightfield; BV, Brilliant Violet; ML, machine learning; SSC, side scatter; RMS, root mean square; ZN, Zombie NIR.

The acquired data from all samples were analyzed using the IDEAS^®^ software (V6.3.41.0, Cytek Bioscience). Multiple .rifs acquired from one all-stained patient sample were merged, and numerous gating steps were applied to identify cells of the megakaryocytic lineage (Figure 2). Visual inspection of the cellular events guided the specific placement of each gate. Initially, to ensure analysis of cells acquired in steady flow, a CD45 versus Time plot was generated, and events in stable flow were selected (Figure 2A). Non-saturated events were gated in each of the used channels as having a Raw Max Pixel value below 4030 (Figure 2B). Next, live and nucleated cells were characterized as negative–low for the viability dye, either ZN or ZG, and as being DRAQ5^+^ (Figure 2C). Lymphocytes were excluded based on their high expression of CD45 and low SSC (Figure 2D) [34]. In a bivariate plot of CD41 and CD3, CD19, CD15, CD64 intensities, cells positive for the megakaryocytic lineage marker were gated while excluding T cells, B cells, granulocytes, and monocytes by gating cells negative for the markers detected in the dump channel (CD3, CD19, CD15, and CD64) (Figure 2E). Next, single cells were identified. The large size of some MKs hindered conventional single cell gating using a bivariate plot of BF Area and BF Aspect Ratio, as large MKs may display feature values similar to those of cell aggregates. Instead, the IDEAS^®^-based machine learning (ML) module (version 6.3.17) was utilized to distinguish single cells from doublets using Linear Discriminant Analysis (LDA). Truth populations were manually tagged (singlets *N* = 28, doublets *N* = 52), and for each object, the ML module generated masks and features based on Ch01 BF and Ch03 CD41 images. LDA was used to generate a linear combination of features that maximized the separation of the two truth populations (Table S1). Then, a classifier-specific value was allocated to each CD41^+^ event and displayed in a histogram, where singlets were gated as cells with a value ≥ 0 (Figure 2F). Events in focus were gated in a histogram displaying BF Gradient Root Mean Square (RMS) (Figure 2G). While this was the last gating step for samples acquired to test the efficacy of MK purification, samples stained with the IFC-MK panel were examined further. A deep learning model (Section 2.8) was applied to select only CD41 membrane-positive cells, ensuring that the CD41 signal originated from the primary cell. Lastly, the diameters of the classified megakaryocytic cells were displayed in a histogram to visually inspect the size range (Figure 2H). All masks and features are described in detail in Figure S1 and Table S2.

### 2.8. Image-Based MK Classification Using Artificial Intelligence

**Figure 3:**
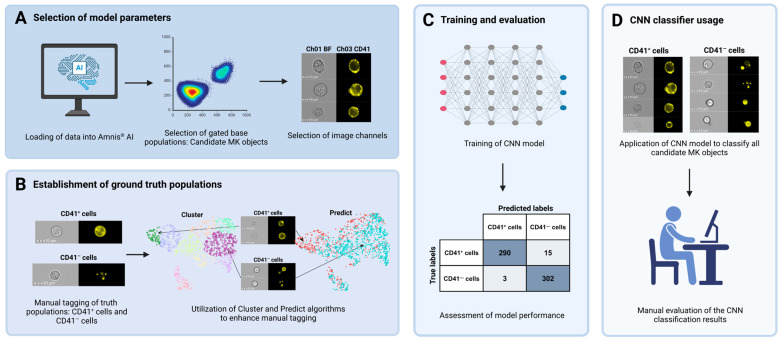
Overview of CNN model development. A CNN model was developed and applied to classify CD41^+^ images captured using IFC as either (I) CD41^+^ cells or (II) CD41^−^ cells with a positive signal derived from adhesive platelets. (**A**) Initially, data files acquired using IFC were gated in the IDEAS^®^ software and loaded into the AAI module. Candidate MK populations were selected from each BM sample, and Ch01 BF and Ch03 CD41 image channels were specified as the basis for classification. (**B**) Ground truth populations were created by a combination of manual tagging and usage of the Cluster and Predict algorithms available through the AAI software, yielding 412 CD41^+^ and 382 CD41^−^ cells. (**C**) The classification model was trained using a CNN algorithm and the training, testing, and validation split technique. Model performance was assessed by examining the results from classification of the testing data, including calculations of various performance metrics. (**D**) Lastly, all candidate MKs gated from each patient sample were classified by application of the developed CNN model, resulting in the distinguishment between CD41^+^ cells and CD41^−^ cells with adhesive platelets. The results were manually assessed by visual evaluation of the classified populations. Abbreviations: AAI, Amnis^®^ Artificial Intelligence; BF, brightfield; BM, bone marrow; CNN, convolutional neural network; IFC, imaging flow cytometry; MK, megakaryocyte.

The Amnis^®^ Artificial Intelligence (AAI) Image Analysis software (V2.0.7, Cytek Biosciences) was used for image-based classification to refine the population of MKs further. This step was necessary because visual inspection of the population obtained using the gating strategy to identify cells of the megakaryocytic lineage (Figure 2) revealed CD41^−^ cells that appeared falsely CD41^+^ due to adhesive platelets. A CNN model was developed to classify cellular images into one of two classes: (I) CD41^+^ cells and (II) CD41^−^ cells (Figure 3). The classification was based solely on Ch01 BF and Ch03 CD41 images. Candidate MK objects, identified as CD41^+^ cells in BM samples from patient ID2 and 10, were imported into the AAI software as the base population. From these, ground truth populations were established through manual tagging and utilization of the Cluster and Predict algorithms included in the AAI software. Ultimately, a total of 794 objects were manually assigned to their corresponding truth model class, constituting 412 CD41^+^ cells and 382 CD41^−^ cells (Figure 3). During CNN model development, the AAI software randomly divided the data into training, validation, and testing sets using a predefined 80/10/10 ratio, where the testing set remained unseen during training [42]. Although an 80/10/10 ratio was stated in the AAI manual, we observed that the data was split using a 76.8/11.6/11.6 ratio. Model training was completed when the accuracy curves of the training and validation sets approached the same value. The model was evaluated by calculation of various performance metrics, including accuracy, precision, recall, and F1 score [42]. Accuracy represents the percentage of correct classifications by calculating the percentage of true predictions (true positives (TPs) plus true negatives (TNs)) out of the total number of events, including TPs, TNs, FPs, and false negatives (FNs):

(1) $$Accuracy=\frac{TP+TN}{TP+TN+FP+FN}$$

Precision expresses the model’s ability to correctly classify positive events:

(2) $$Precision=\frac{TP}{TP+FP}$$

Recall, also known as sensitivity or the true positive rate, indicates the fraction of true positive events that were classified as such:

(3) $$Recall=\frac{TP}{TP+FN}$$

Lastly, the F1 score is defined as the harmonic mean of recall and precision:

(4) $$F_{1}=\frac{2\cdot precision\cdot recall}{precision+recall}$$

The established CNN classifier was used to classify candidate MKs from all patient samples (ID2–10), gated according to the gating strategy depicted in Figure 2.

A manual evaluation of the CNN classification results was performed, encompassing visual confirmation of the results from ID5 and ID7 (*N* = 441).

## 3. Results

### 3.1. CD41 Positive Selection Ensured Enrichment of Megakaryocytic Cells

**Figure 4:**
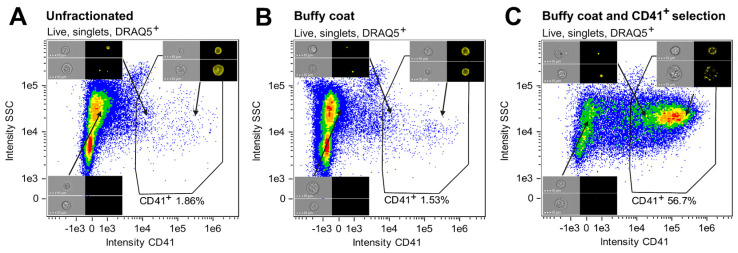
Proportions of CD41^+^ cells at specific steps of the CD41^+^ cell enrichment procedure. The proportions of CD41^+^ cells are given as percentages of live, nucleated singlets in (**A**) unfractionated cells, (**B**) buffy coat-isolated material, and (**C**) cells subjected to buffy coat isolation and CD41 positive selection. Representative Ch01 BF and Ch03 CD41 images of CD41^−^ and CD41^+^ cells are included. Abbreviations: MK, megakaryocyte; SSC, side scatter.

A protocol for enriching cells of the megakaryocytic lineage based on their CD41 expression was established, incorporating isolation of nucleated cells and CD41 positive selection (Figure 1). The enrichment of CD41^+^ cells at specific steps in the procedure was evaluated by analyzing three fractions created from BM ID1: (I) unfractionated BM, (II) cells collected from the buffy coat, and (III) cells obtained after buffy coat isolation and CD41 positive selection. While CD41^+^ cells only constituted 1.86% of the unfractionated sample, the sample subjected to both buffy coat isolation and CD41 positive selection contained 56.7% CD41^+^ cells, showing a high degree of enrichment for CD41^+^ cells (Figure 4A, C). On the other hand, the percentage of CD41^+^ cells following only buffy coat isolation (Figure 4B) was comparable to that of the unfractionated sample (Figure 4A).

Using a BM sample from ID11, we assessed the CD41 positive enrichment procedure, confirmed the presence of MKs (Figure S2A), and demonstrated that the majority of MKs were retained within the selected CD41^+^ cell fraction. Specifically, MKs at various stages of differentiation were observed, with a median diameter of 22.5 µm (range: 10.5–51.5 µm) and varying ploidy levels (Figure S2B,C). The majority of MKs were retained in the CD41-enriched sample (Figure 2D). Thus, although some loss of MKs during the washing steps is unavoidable, only a limited number of MKs were detected in the supernatants, indicating efficient retention of MKs in the enriched fraction.

**Figure 5:**
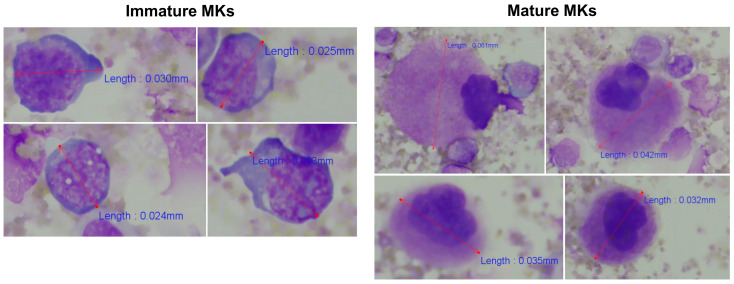
Identification of immature and mature MKs among BM cells after CD41 positive selection. The morphological examination identified both mature and immature MKs. MKs were recognized as large cells with lobulated nuclei and abundant, finely granular cytoplasm [20], whereas the more immature MKs were identified as smaller non-lobulated or bilobed cells with a higher nucleocytoplasmic ratio and immature chromatin pattern [43]. Cells were evaluated and counted using 400x magnification. Representative cells and their length measurements are shown. Abbreviations: BM, bone marrow; MK, megakaryocyte.

As a quality control measure of the established protocol for enrichment of CD41^+^ cells (Figure 1), the percentages of MKs in unfractionated BM and in CD41^+^ selected cells were estimated and compared by microscopy. In the diagnostic BM aspirate smear from patient ID10, only one MK was observed among 8261 nucleated cells (approximately 0.0001%). In contrast after CD41 positive selection, a total of 84 MKs were identified while assessing 463 nucleated cells (approximately 18%). Interestingly, CD41^+^ cell selection enabled recognition of the usually less frequently identified MK precursors, i.e., immature MKs [20], along with mature MKs (Figure 5), suggesting the potential of this procedure to assess multiple stages of megakaryopoiesis.

### 3.2. Combination of IFC and Image-Based Classification Enabled Effective Identification of MKs

**Figure 6:**
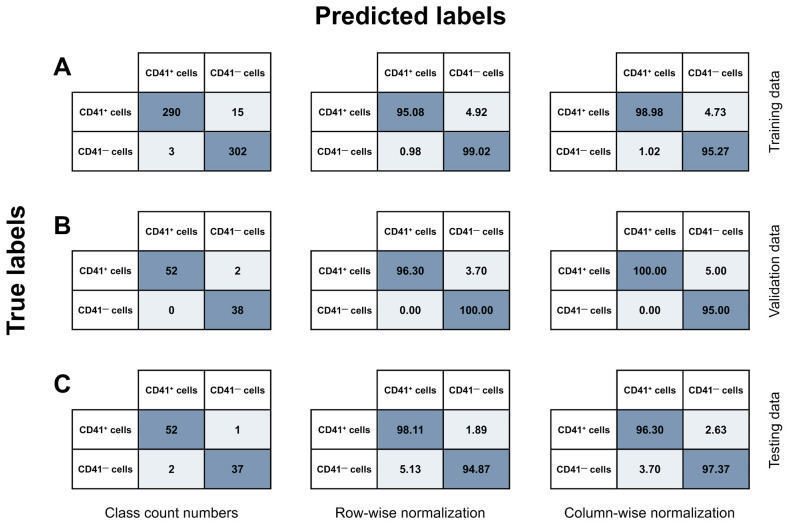
Confusion matrices. A CNN model was trained and utilized to classify a total of 794 cells into two categories: (I) CD41^+^ cells and (II) CD41^−^ cells, based on Ch01 BF and Ch03 CD41 images captured on the ISX. All events were candidate MK objects from two BM samples subjected to CD41^+^ cell selection to enrich for cells of the megakaryocytic lineage. The data were split into (**A**) training (*N* = 610), (**B**) validation (*N* = 92), and (**C**) testing (*N* = 92) data. The CNN model results were recorded in confusion matrices, showing the relationship between true and predicted labels. The results are shown as class count numbers (**left panel**); row-wise normalization, showing recall for each class (**middle panel**); and column-wise normalization, signifying precision for each class (**right panel**). Abbreviations: BF, brightfield; BM, bone marrow; FN, false negative; FP, false positive; ISX, ImageStream^®X^ Mk II; TN, true negative; TP, true positive; MK, megakaryocyte.

A five-color IFC panel including eight markers (IFC-MK panel, Table 3) was successfully designed and utilized to identify MKs among CD41^+^ BM cells by combining immunophenotyping and image-based morphometric analysis. Initially, the immunophenotypes of cells of the megakaryocytic lineage were used to carry out a series of gating steps leading to the population of interest (Figure 2). In brief, images of live, nucleated, non-lymphocytes in stable flow were identified (Figure 2A–D). Then, cells of the megakaryocytic lineage were gated as CD41^+^CD3^−^CD19^−^CD15^−^CD64^−^ (Figure 2E), and singlets in focus were selected as having ML classifier values ≥ 0 and a gradient RMS > 55 (Figure 2F, G). A visual inspection of the IFC imagery revealed that the population of megakaryocytic cells included cells with a CD41^+^ membrane (i.e., TP events), as well as CD41^−^ cells that displayed CD41 positivity due to adhesive platelets (i.e., FP events). For TP CD41^+^ cells, the CD41 signal appeared as a spotted pattern with multiple individual spots distributed along the cell perimeter, whereas FP cells showed only a few CD41^+^ spots originating from platelets adhering to the cells (Figure 3B, D). To maximize removal of FP MKs, a deep learning model employing a CNN algorithm was successfully developed to classify events into two classes: (I) CD41^+^ cells and (II) CD41^−^ cells (Figure 3), only based on BF and CD41 images captured with IFC, leading to a final classified CD41^+^ MK population (Figure 2H). After model training, various evaluation metrics were calculated, including accuracy, precision, recall, and F1 score, obtained from the training, validation, and testing data (Table 4). The statistics varied from 95.0% to 100%, with overall weighted average F1 scores of 97.0%, 97.8%, and 96.7% for the training, validation, and testing datasets, respectively (Table 4). Additionally, true and predicted class values were recorded in confusion matrices (Figure 6).

Based on the testing data (Figure 6C, left panel), the overall accuracy of the CNN model was 96.74%, showing substantial AI-detected image differences between CD41^+^ and CD41^−^ cells and the ability of the model to effectively differentiate between the two model classes. The model demonstrated a high sensitivity, as it correctly retrieved 98.11% of the true CD41^+^ MKs (Figure 6C, middle panel). Furthermore, with respect to the accurate identification of TPs, the model showed high precision, successfully classifying CD41^+^ cells as positives in 96.30% of cases (Figure 6C, right panel). Only 1.89% of true CD41^+^ cells were misclassified as CD41^−^ cells, while 5.13% of CD41^−^ cells were incorrectly categorized as CD41^+^ MKs.

**Figure 7:**
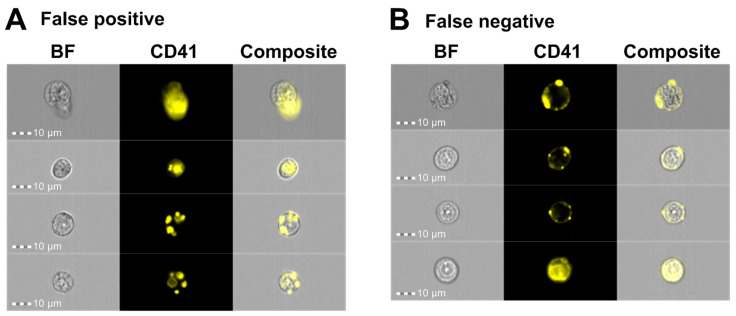
IFC imagery examples of cells misclassified by the AAI CNN model. Representative images of (**A**) false positive and (**B**) false negative events following CNN classification of gated CD41^+^ events using Ch01 BF and Ch03 CD41 images captured on the ISX. Abbreviations: AAI, Amnis^®^ Artificial Intelligence; BF, brightfield; BM, bone marrow; CNN, convolutional neural network; FN, false negative; FP, false positive; IFC, imaging flow cytometry; ISX, ImageStream^®X^ Mk II.

Next, we performed manual validation by visually inspecting the images after applying the CNN classification model (Figure 3D) to classify 185 CD41^+^ events from ID5 and 256 CD41^+^ events gated from ID7. The classification results revealed that the model correctly retrieved 90.4% and 87.0% of the true CD41^+^ cells for ID5 and ID7, respectively (Table 5). Among the true CD41^−^ cells, 92.2% and 85.0% could be correctly retrieved. Out of all events predicted as CD41^+^, 89.2% were correct for ID5 and 84.3% for ID7, whereas the corresponding numbers for CD41^−^ cells were 93.1% and 87.6% (Table 5). Visual inspection of the CD41^−^ cells misclassified as CD41^+^ cells specified that their CD41 signal covered a substantial part of the primary cell (Figure 7A). However, the CD41^+^ signal predominantly originated from platelets that were either captured in a different focal plane than the primary cell, thereby blurring the CD41 image and increasing the number of pixels capturing a CD41^+^ signal, or from multiple platelets adhering to a CD41^−^ cell, forming an almost complete ring of scattered CD41^+^ platelets (Figure 7A). By comparison, the images of CD41^+^ cells misclassified as CD41^−^ included events with very bright CD41^+^ platelet signals that nearly masked the weaker CD41^+^ membrane signal, as well as events captured at an angle that displayed the CD41^+^ platelet positioned on top of the primary cell (Figure 7B).

**Figure 8:**
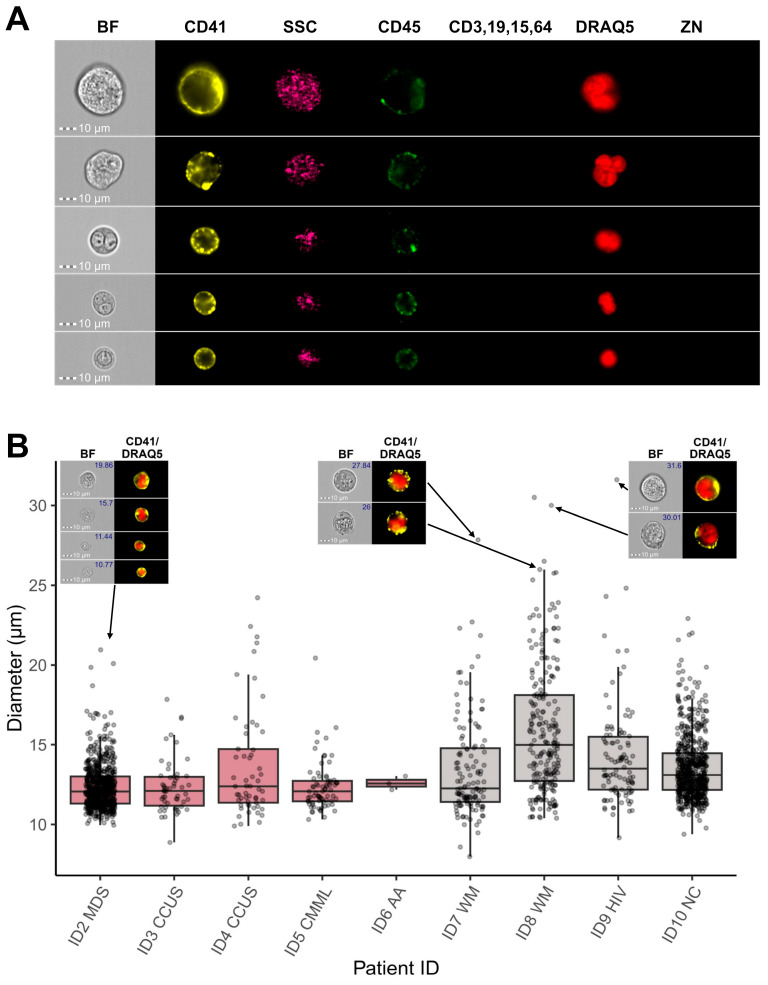
Megakaryocytic cells identified by IFC. (**A**) Representative images of BM MKs identified by IFC-specific gating, including immunophenotyping and image-based morphometrics, in combination with CNN-based image classification. Each row shows one single cell, while columns display images obtained in different channels on the ISX. (**B**) Boxplots showing the diameter values in µm of MKs gated from each patient sample. The horizontal lines indicate the first, second, and third quartiles. Representative Ch01 BF and composite Ch03 CD41/Ch11 DRAQ5 images are shown for MKs with different sizes. Diameter values are indicated in the top right of BF images. Abbreviations: AA, aplastic anemia; BF, brightfield; BM, bone marrow; CCUS, clonal cytopenia of undetermined significance; CMML, chronic myelocytic leukemia; CNN, convolutional neural network; HIV, human immunodeficiency syndrome; IFC, imaging flow cytometry; ISX, ImageStream^®X^ Mk II; MDS, myelodysplastic neoplasms; MKs, megakaryocytes; NC, not conclusive; WM, Waldenstrom macroglobulinemia; ZN, Zombie NIR.

Based on these results, the CNN classification model was applied as the final gating step to all patient samples, and incorporating CNN-based image classification into the gating strategy facilitated the exclusion of the majority of FP MKs from subsequent analyses, thereby refining the final population of true megakaryocytic cells (Figure 8A). MKs of various sizes could be detected in the BM samples from different patients, with MK diameters ranging from <10 µm to >30 µm (Figure 8B). Ultimately, the combination of IFC-specific gating and CNN-based image classification enables accurate identification and quantification of microMKs in BM aspirate samples.

## 4. Discussion

Accurate identification and characterization of MKs and their progenitors remain particularly challenging. Their low abundance and complex morphology complicate reliable quantitative analysis and reduce sensitivity, especially in microscopy. While conventional flow cytometry has shown promise due to its high-throughput capacity, thereby enabling analysis of rare cell types within heterogenous samples, issues arise when platelets adhere to non-MK cells, causing these cells to display an MK-like immunophenotype [24,33]. Furthermore, due to their substantial size and complex morphology, the largest MKs often exceed the forward scatter (FSC) detection range on a linear scale presentation, leading to off-scale events that risk being excluded during the initial FSC/SSC gating of live leukocytes [44,45]. Here, we introduce a novel approach for identification of megakaryocytic cells that combines selective enrichment of CD41^+^ cells (Figure 1) with IFC, utilizing image data for MK identification in human BM samples (Figure 2, Figure 3, and Figure S2). Previous studies have demonstrated the use of IFC to identify MKs within heterogeneous murine BM samples [38,39,46]; however, to the best of our knowledge, our study presents the first technique for MK enrichment and effective identification of microMKs in human BM. Unique to the IFC technique, the ability to analyze images of every single cell while assessing surface marker expression as well as the fluorescence signal location and origin provides a valuable tool for visual verification and refinement of gating strategies (Figure 2). Moreover, IFC does not include FSC but instead the cell area and shape may be used for single cell gating, and any object within the field of view, as defined by the selected objective lens, will be captured. In the context of MKs, we encountered significant challenges using the classic singlet cell gating based on area, as large MKs may overlap with doublets, triplets, or even multiplets that exhibit similar Area and Aspect Ratio characteristics. Yet, by extracting a wide range of features from the high-resolution images, detailed characterization of both singlets and doublets can be achieved, and a linear discriminant combination of features can be incorporated into a classifier (Figure 2, Table S1) to enhance the separation between these populations, thereby improving the accuracy of distinguishing true singlets from doublets or cell aggregates. Although the ISX provides a maximum of 60x magnification, leading to a reduced resolution compared to what can be achieved with traditional light microscopy (e.g., 400–1200x), the IFC-based analysis benefits from examining a much larger proportion of the BM aspirate in an objective manner. However, the considerable variation in both size and ploidy levels among MKs [6,8] present challenges for simultaneously detecting all MK maturation stages using the IFC technique. In this study, we focused on optimizing the panel to enable the reliable identification of microMKs, a subset of abnormally small megakaryocytes (7–15 µm in diameter) characterized by dense chromatin and a limited cytoplasmic volume and frequently observed in MDS [47]. A key priority was achieving high image resolution to ensure accurate masking of cell and nuclear regions, allowing for morphometric assessments such as size. For this purpose, we employed the 40x objective with a 60 µm field of view and a pixel size of 0.5 µm, which provided a significantly higher image resolution compared to the 20x objective (120 µm field of view, 1 µm pixel size). Although the 20x objective would allow for detection of the largest MKs, the superior resolution obtained with the 40x objective was critical for capturing more detailed images of the smaller cells. Furthermore, this magnification nevertheless facilitated the detection of MKs with diameters ranging from 10.5 to 51.5 µm (Figure S2), encompassing the majority of human BM MKs in suspension, which have been reported to range from 12 to 63 µm in diameter [48]. Of note, detecting large MKs necessitates adjustment of the laser power settings and CD41 antibody concentrations to avoid pixel saturation.

Utilizing a combination of immunophenotypic markers (Table 3) and morphometry-based gating enabled partial exclusion of non-MKs (Figure 2). Ultimately, a CNN algorithm was trained and utilized to distinguish true MKs from non-MKs displaying an MK-like immunophenotype due to adhesive CD41^+^ platelets (Figure 3). Accuracy metrics demonstrated that this approach effectively enabled removal of 94.9% of the residual FP MKs (Table 4, Figure 6), yielding a refined MK subset (Figure 8). The model demonstrated high precision and recall, correctly classifying MKs in 96.3% of cases and identifying 98.1% of true MKs in the testing dataset (Table 4). Clinically, this means that labeling an event as an MK indicates a high level of confidence in its accuracy, with only a small portion (1.9%) of true MKs being misclassified as non-MKs. This approach, therefore, enables identification of nearly all true MKs with high precision. Though the number of FP MKs may vary between patient samples, the value of their removal is evident, enhancing specificity over conventional flow cytometry by enabling reliable exclusion of CD41^+^ non-MKs from the analysis. Moreover, the number of FP and FN MKs could potentially be decreased through further improvement of the CNN classification, e.g., by including a model class retrieving these specific events. Building on insights from previous studies, this highlights the significant advantage of integrating immunophenotyping with AI-based image analysis for detection of morphological differences [42,49,50,51,52,53].

Morphological examination of dysmegakaryopoiesis is a fundamental part of the diagnostic evaluation of BM samples from patients suspected of having MDS, however, accurate assessment of dysplastic changes demands significant expertise and is subject to considerable inter-observer variability [14,15,16,17]. Morphological assessment of 30 MKs is recommended as the basis for evaluation [18,19,20,21], although reaching this number can be challenging due to their low frequency, especially in inadequate samples. It is widely recognized that increasing the number of analyzed cells improves the reproducibility of any dysplasia assessment [54]. The IFC technology is particularly well-suited for this purpose, as it enables the acquisition of virtually unlimited cell numbers while imaging every single cell and incorporating phenotypic markers to target specific cell lines. With the automated acquisition and analysis templates integral to the technology, the IFC approach supports the objective and systematic quantification of microMKs, including morphometric measurements (Figure 8B), effectively overcoming the limitations of traditional microscopy, such as subjectivity, inter-observer variability, and the time-consuming nature of manual evaluation [14,15,16,17]. Although the CD41 positive selection purity was only around 50%, the MK frequency was increased nearly 200-fold (Figure 4), significantly reducing the acquisition time. For the IFC-acquired samples, more than 1800 MKs were collected across nine patients, allowing analysis of a substantially larger portion of BM material, thus enhancing the statistical robustness and sensitivity of future MK and microMK analyses. Incorporating the CD41 antigen into the IFC-MK panel (Table 3) ensured inclusion of all stages of MK development [6,8], while the addition of a dump channel enabled exclusion of CD41^+^ non-MKs (CD41 positivity caused by adhesive CD41^+^ platelets or true CD41 membrane expression among dump^+^ cells), resulting in a refined MK population. Moreover, examining the enriched sample manually through microscopy established the presence of more immature megakaryocyte progenitors in addition to mature MKs (Figure 5), indicating the potential of this procedure to evaluate early megakaryopoietic progenitor stages.

In summary, this study validates the use of a simple CD41 positive cell selection procedure combined with an IFC antibody panel for effective enrichment of MKs and identification of microMKs. Additionally, we demonstrate the importance of incorporating a CNN-based algorithm to enhance specificity by excluding FP MKs from the analysis. Thus, we put forward an automated template for the systematic study of microMKs, a rare cell type that has been exceedingly difficult to analyze. This work highlights the advantages of IFC for MK-related analyses, suggesting that the application of this method in a clinical setting with impaired MK development could improve the objective and quantitative analysis of MKs and their progenitors.

## Figures and Tables

**Table 1:** Patients.

Patient ID	Age	Gender	Diagnosis
1	71	M	MDS
2	79	M	MDS
3	81	M	CCUS
4	44	M	CCUS
5	75	M	CMML
6	54	M	AA
7	74	M	WM
8	77	F	WM
9	59	M	HIV
10	87	M	NC
11	24	M	Infection

Abbreviations: AA, aplastic anemia; CCUS, clonal cytopenia of unknown significance; CMML, chronic myelomonocytic leukemia; HIV, human immunodeficiency virus; MDS, myelodysplastic neoplasms; NC, not conclusive; WM, Waldenström’s macroglobulinemia.

**Table 2:** Antibodies and dyes used to evaluate the effects of CD41 positive enrichment.

Specificity	Fluorophore/Stain	Clone	Vendor	Cat #	Titer	Quantity *
CD41	PE	MWReg30	BioLegend	133905	1:20	5 µL
CD45	KrO	J33	Beckman Coulter	PN A966416	1:40	2.5 µL
DNA	DRAQ5	NA	eBioScience	65-0880-92	NA	2.5 µM
Viability	ZG	NA	BioLegend	423112	1:200	0.5 µL

* Volumes are specified as μL/100 μL sample. Antibodies and dyes were supplied by BioLegend (San Diego, CA, USA), Beckman Coulter (Brea, CA, USA), and eBioScience (San Diego, CA, USA). Abbreviations: Cat, catalogue; KrO, Krome Orange; NA, not applicable; PE, phycoerythrin; ZG, Zombie Green.

**Table 3:** Antibodies and dyes included in the IFC-MK panel.

Specificity	Fluorophore/Stain	Clone	Vendor	Cat #	Titer	Quantity *
CD41	PE	HIP8	BioLegend	303706	NA	3 µg/mL
CD45	SBV515	F10-89-4	Bio-Rad Laboratories	MCA87SBV515	1:20	5 µL
CD3	BV605	SK7	BioLegend	344836	1:80	1.25 µL
CD19	BV605	HIB19	BioLegend	302244	1:160	0.625 µL
CD15	BV605	W6D3	BioLegend	323032	1:40	2.5 µL
CD64	BV605	10.1	BioLegend	305034	1:40	2.5 µL
DNA	DRAQ5	NA	eBioScience	65-0880-92	NA	1.25 µM
Viability	ZN	NA	BioLegend	423105	1:400	0.25 µL

* Volumes are specified as μL/100 μL sample. Antibodies and dyes were supplied by BioLegend (San Diego, CA, USA), Bio-Rad Laboratories (Hercules, CA, USA), and eBioScience (San Diego, CA, USA). Abbreviations: BV, Brilliant Violet; Cat, catalogue; NA, not applicable; PE, phycoerythrin; SBV, StarBright Violet; ZN, Zombie NIR.

**Table 4:** Accuracy statistics for the training, validation, and testing data used in the AAI CNN model.

Model Class	Training Data				Validation Data				Testing Data			
	Objects (*N*)	Precision (%)	Recall (%)	F1 (%)	Objects (*N*)	Precision (%)	Recall (%)	F1 (%)	Objects (*N*)	Precision (%)	Recall (%)	F1 (%)
**CD41^+^** **cells**	305	99.0	95.1	97.0	54	100.0	96.3	98.1	53	96.3	98.1	97.2
**CD41^−^** **cells**	305	95.3	99.0	97.1	38	95.0	100.0	97.4	39	97.4	94.9	96.1
**Weighted** **average**	610	97.1	97.0	97.0	92	97.9	97.8	97.8	92	96.8	96.7	96.7

A CNN classifier was trained to discriminate CD41^+^ and CD41^−^ cells. This model was based on BF and CD41 images captured in Ch01 and Ch03, respectively, on the ISX. The performance of the model was evaluated by calculating precision, recall, and F1 scores for each data set. Abbreviations: AAI, Amnis^®^ Artificial Intelligence; BF, brightfield; CNN, convolutional neural network; ISX, ImageStream^®X^ Mk II.

**Table 5:** Accuracy statistics for manual validation of the AAI CNN model.

Model Class	Manual Evaluation							
	ID5 CMML				ID7 WM			
	Objects (*N*)	Precision (%)	Recall (%)	F1 (%)	Objects (*N*)	Precision (%)	Recall (%)	F1 (%)
**CD41^+^ cells**	83	89.2	90.4	89.8	123	84.3	87.0	85.6
**CD41^−^ cells**	102	93.1	92.2	92.6	133	87.6	85.0	86.3
**Weighted average**	185	91.1	91.3	91.2	256	85.9	86.0	85.9

A CNN classifier was trained to discriminate CD41^+^ and CD41^−^ cells and subsequently utilized to classify 185 events gated from ID5 and 256 events gated from ID7 based on their Ch01 BF and Ch03 CD41 images. The results were manually evaluated by visual examination of the images. The performance of the model was evaluated separately for ID5 and ID7 by calculating precision, recall, and F1 scores. Abbreviations: AAI, Amnis^®^ Artificial Intelligence; BF, brightfield; CMML, chronic myelomonocytic leukemia; CNN, convolutional neural network; WM, Waldenstrom macroglobulinemia.

## Data Availability

The data presented in this study are available upon reasonable request from corresponding author C.A.R.

## Supplementary Materials

The following supporting information can be downloaded at https://www.mdpi.com/article/10.3390/cells14080588/s1, Figure S1. Masking strategy; Figure S2. Evaluation of the CD41 positive enrichment procedure; Table S1. Features included in the Linear Discriminant Analysis ML singlets classifier; and Table S2. Feature list.
