# Supplementary material for: High-Efficiency Enrichment of Megakaryocytes and Identification of Micromegakaryocytes from Human Bone Marrow by Imaging Flow Cytometry
Source: Cells. 2025 Apr 12;14(8):588. doi: 10.3390/cells14080588 (PMC12026402; doi:10.3390/cells14080588)
Supplement: Supplementary file 1 [file cells-14-00588-s001.zip › cells-3365810-supplementary.pdf]

cells MDPI

Supplementary Data

High-efficiency enrichment of megakaryocytes and identification of micromegakaryocytes from human bone marrow by imaging flow cytometry, Pedersen & Hybel et al. 2025, Cells

A

CELLULAR REGION		
**Mask name**	**Description**	**BF / Mask**
M01, Ch01 BF	Default mask capturing the signal brighter than the background in the image. Highlights the cellular region in an inclusive manner.	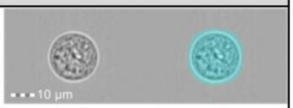
Object(M01, Ch01 BF, tight)	Refinement of the cellular mask to the shape of the image. Provides a tighter fit compared to the default cell mask, thus enabling a more precise circularity measurement.	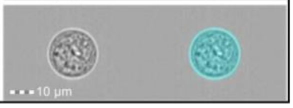

B

CD41 REGION		
**Mask name**	**Description**	**BF / CD41 / Mask**
M03, Ch03 CD41	Default mask designed to capture the signal brighter than the background in the image.	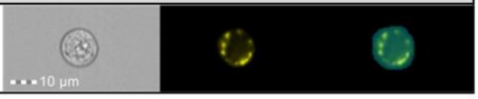

C

NUCLEUS		
**Mask name**	**Description**	**BF / DRAQ5 / Mask**
MC, Ch11 DRAQ5	Combined mask created by fusion of the masks highlighting pixels detected as brighter than the background in all channels. Used to select the nuclear area of each cell.	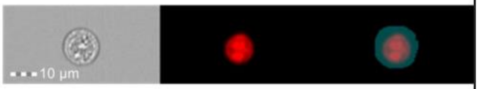

D

FLUORESCENCE		
**Mask name**	**Description**	**BF / Fluorescence / Mask**
MC, Ch03 CD41 PE	Combined mask created by fusion of the masks highlighting pixels detected as brighter than the background in all channels. Used to mask the fluorescence detected from fluorophores/dyes.	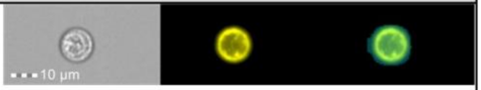
MC, Ch08 CD45 SBV515		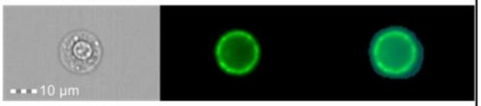
MC, Ch10 CD3, 19, 15, 64 BV605		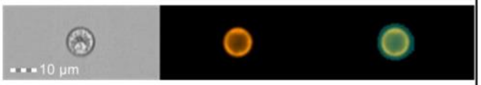
MC, Ch12 ZN		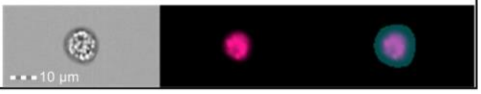

E

GRANULARITY		
**Mask name**	**Description**	**BF / SSC / Mask**
MC, Ch06 SSC	Combined mask created by fusion of the masks highlighting pixels detected as brighter than the background in all channels. Used to mask SSC signals and thereby the granular areas of each cell.	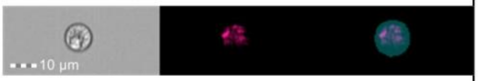

Figure S1: Masking strategy. Masking strategy and purpose of the masks incorporated in the gating strategy for identification of MKs. **A.** The cellular image in the BF channel was masked in an inclusive manner using the default M01 mask. When measuring Circularity, the Object mask was applied instead, removing excess background pixels and thereby providing a tighter fit, ensuring precise quantification. **B.** The default M03 mask was used to mask CD41^{+} signals in an inclusive manner by detecting all pixels brighter than the background. **C.-E.** The MC mask, which merges all masks highlighting pixels brighter than the background signals in all channels, was utilized to capture the nuclear area (DRAQ5), fluorescence signals from fluorophores/dyes (CD41 PE, CD45 SBV515, CD3, CD19, CD15, and CD64 BV605, ZN), and granular areas of the cells (SSC). Abbreviations: BF; brightfield, BV; Brilliant Violet, Ch; channel, MC; Combined Mask, PE; phycoerythrin, SBV; StarBright Violet, SSC; side scatter, ZN; Zombie NIR.

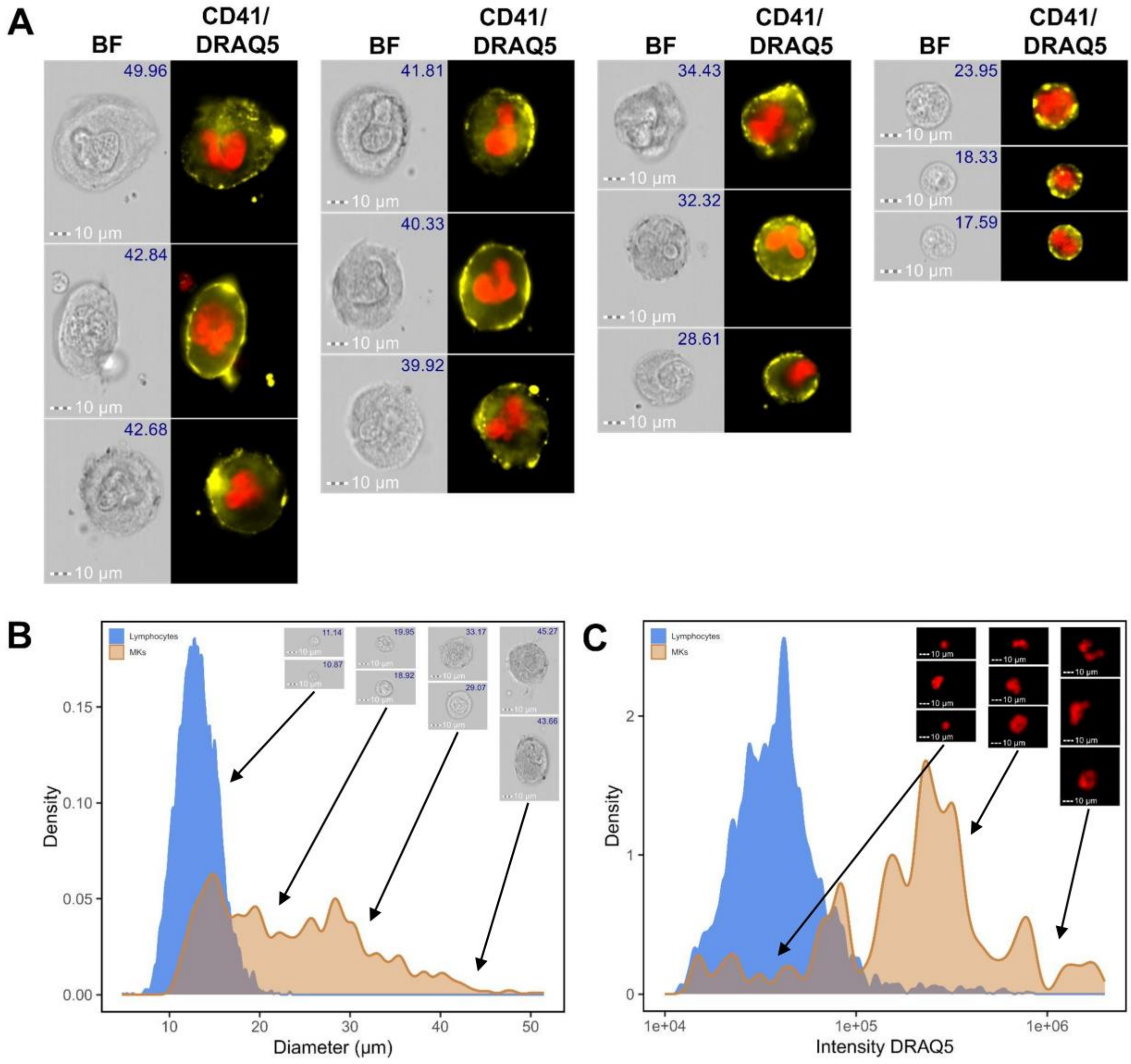

D

Sample	Number of MKs (frequency*)	
	Diameter > 20 μm	Diameter < 20 μm
**CD41-enriched**	420 (0.21%)	295 (0.148%)
**Supernatant 1**	51 (0.010%)	57 (0.011%)
**Supernatant 2**	153 (0.031%)	73 (0.015%)
**Supernatant 3**	20 (0.015%)	16 (0.012%)

Figure S2: Evaluation of the CD41 positive enrichment procedure. A BM sample from patient ID11 was subjected to CD41^{+} enrichment by three rounds of magnetic washing. The CD41-enriched eluate was collected as well as the supernatant from each washing step. Samples were stained with the IFC-MK panel (CD41 PE concentration adjusted to 1.5 μg/mL) and acquired using the ISX. MKs were subsequently identified through manual tagging of CD41^{+} cells and lymphocytes were gated as CD45^{high}SSC^{low}. **A.** Representative Ch01 BF and composite Ch03 CD41/Ch11 DRAQ5 images are shown of MKs of various sizes identified within the CD41-enriched sample. **B.** Composite density histogram visualization of the range in diameters in μm of MKs (orange) and lymphocytes (blue), showing the presence of MKs at various differentiation stages within the CD41-enriched sample, ranging from small immature MKs to large, mature MKs. Representative Ch01 BF images are shown for both lymphocytes and MKs of various sizes. Specific diameter values for each cell are shown in the top right corners of BF images in A and B. **C.** Composite density histogram representation of Ch11 DRAQ5 signal intensity for MKs (orange) compared with lymphocytes (blue), demonstrating the range in ploidy levels within

the MK population. Representative Ch11 DRAQ5 images are shown of MKs with different DNA contents. **D.** Table showing the number and frequency of large (> 20 µm) and small (< 20 µm) CD41⁺ MKs within both the CD41-enriched eluate as well as the supernatant retained following each round of magnetic washing, illustrating that while few MKs are lost in the supernatant during the washing procedure, most are retained within the CD41-enriched eluate. *The frequency of MKs was calculated as the percentage of all events acquired from each sample. Abbreviations: BF; brightfield, BM; bone marrow, IFC; imaging flow cytometry, ISX; ImageStream®X Mk II, MK; megakaryocyte, SSC: side scatter.

Table S1: Features included in the Linear Discriminant Analysis ML singlets classifier.

Mask	Feature	Weight
**Ch01 BF**		
M01	Aspect Ratio	6.15
	Circularity	4.61
	Elongatedness	-4.82
	Shape Ratio	4.63
Morphology	Aspect Ratio	6.15
	Circularity	4.61
	Elongatedness	-4.82
	Shape Ratio	4.62
	Symmetry 2	-5.05
	Symmetry 4	-4.59
Object, tight	Aspect Ratio	6.41
	Circularity	5.14
	Shape Ratio	5.08
	Symmetry 2	-4.75
Object, inclusive	Circularity	5.03
Skeleton (Object, tight), thick	Symmetry 2	-4.71
Skeleton, thick	Symmetry 3	-4.61
Skeleton (Morphology), thick	Symmetry 3	-4.61
**Ch06 SSC**		
Object, tight	Shape Ratio	4.74
	Thickness Min	4.86

Features included in the super-feature created to distinguish between doublets and singlets using the Linear Discriminant Analysis algorithm in the IDEAS-based ML module. Masks and features were generated, ranked and combined during training based on manually tagged truth populations. Abbreviations: BF; brightfield, ML; machine learning, SSC; side scatter.

Table S2: Feature list.

Category	Feature	Definition	Usage
**System**	Time	Camera timer values converted to seconds.	Gating of cells in stable flow.
**Signal strength**	Intensity	Sum of the pixel intensities within the selected mask, background subtracted.	Gating of specific cell populations
	Raw Max Pixel	Largest value of pixel intensity within the selected mask.	Exclusion of images containing pixels out of range.
**Size**	Diameter	The diameter of a circle with an area equal to that of the object.	Assessment of the approximate cell size.
**Texture**	Gradient Root Mean Square	Measures the focus quality of an image by the changes of pixel values within the selected mask.	Gating of images in focus.

List of features and their specific usage during the gating strategy performed to identify MKs in the IDEAS software.
